# *Acinetobacter baumannii* maintains its virulence after long-time starvation

**DOI:** 10.1371/journal.pone.0201961

**Published:** 2018-08-22

**Authors:** Itziar Chapartegui-González, María Lázaro-Díez, Zaloa Bravo, Jesús Navas, José M. Icardo, José Ramos-Vivas

**Affiliations:** 1 Instituto de Investigación Valdecilla IDIVAL, Santander, Spain; 2 Servicio de Microbiología, Hospital Universitario Marqués de Valdecilla, Santander, Spain; 3 Red Española de Investigación en Patología Infecciosa (REIPI), Instituto de Salud Carlos III, Madrid, Spain; 4 Departamento de Biología Molecular, Universidad de Cantabria, Santander, Spain; 5 Departamento de Anatomía y Biología Celular, Universidad de Cantabria, Santander, Spain; Wadsworth Center, UNITED STATES

## Abstract

*Acinetobacter baumannii* is a cause of healthcare-associated infections. Although *A*. *baumannii* is an opportunistic pathogen, its infections are notoriously difficult to treat due to intrinsic and acquired antimicrobial resistance, often limiting effective therapeutic options. *A*. *baumannii* can survive for long periods in the hospital environment, particularly on inanimate surfaces. Such environments may act as a reservoir for cross-colonization and infection outbreaks and should be considered a substantial factor in infection control practices. Moreover, clothing of healthcare personnel and gadgets may play a role in the spread of nosocomial bacteria. A link between contamination of hospital surfaces and *A*. *baumannii* infections or between its persistence in the environment and its virulence has not yet been established. Bacteria under stress (i.e., long-term desiccation in hospital setting) could conserve factors that favor infection. To investigate whether desiccation and/or starvation may be involved in the ability of certain strains of *A*. *baumannii* to retain virulence factors, we have studied five well-characterized clinical isolates of *A*. *baumannii* for which survival times were determined under simulated hospital conditions. Despite a considerable reduction in the culturability over time (up to 88% depending on strain and the condition tested), some *A*. *baumannii* strains were able to maintain their ability to form biofilms after rehydration, addition of nutrients, and changing temperature. Also, after long-term desiccation, several clinical strains were able to grow in the presence of non-immune human serum as fine as their non-stressed homologs. Furthermore, we also show that the ability of bacterial strains to kill *Galleria mellonella* larvae does not change although *A*. *baumannii* cells were stressed by long-term starvation (up to 60 days). This means that *A*. *baumannii* can undergo a rapid adaptation to both the temperature shift and nutrients availability, conditions that can be easily found by bacteria in a new patient in the hospital setting.

## Introduction

*A*. *baumannii* is a non-motile opportunistic extracellular human pathogen. Antibiotic-resistant *A*. *baumannii* has emerged as one of the most problematic nosocomial pathogens [1]. While more than 80 complete genome sequences of several strains of *A*. *baumannii* have been published, only very few potential virulence factors have been implicated in its disease pathogenesis [2]. The outer membrane protein (OmpA) and a capsular polysaccharide seem to be involved in the interaction with epithelial cells *in vitro* or in virulence [3, 4]. Other surface components or secreted proteins play a minor role both *in vitro* or *in vivo*. With these limited number of virulence factors, it has been suggested that the fulminant course of disease might be due to exaggerated host response to *A*. *baumannii* lipopolysaccharide [5–8].

As with other non-fermentative Gram-negative bacilli, *A*. *baumannii* can develop resistance to all classes of antimicrobials; and multi-drug resistant (MDR) isolates are sharply increasing in frequency, forcing clinicians to use last resort antibiotics such as colistin [9–11]. Several studies showed the presence of this pathogen in various hospital environments where *A*. *baumannii* is transmitted by direct contact with infected patients or indirect contact with contaminated inanimate surfaces [12–14]. Importantly, cross-transmission of microorganisms from abiotic surfaces may have a significant role in ICU-acquired infections [15–17].

One important factor contributing to the spread of *A*. *baumannii* in these environments seems to be its capacity to withstand desiccation and starvation [18–20]. Also, it can produce biofilms, a community of bacteria enclosed within a protective polymeric matrix [21]. This ability to form biofilms is another potential virulence factor because it increases the survival rate of this bacterium on dry surfaces and may contribute to its persistence in the hospital environment, increasing the probability of causing nosocomial infections and outbreaks [1].

Despite the link among contamination, *Acinetobacter* survival in the patient care environment and the risk of healthcare-associated infections have not yet been established, gaining insight into the mechanisms of long-term persistence of this pathogen in hospital settings is fundamental to prevent clonal spread, and to the development of novel targets for both diagnostic tests and therapeutic agents. There is a knowledge gap concerning the bacterial transition from a stressed state (i.e., on inanimate surfaces) to a new environment with available nutrients and higher temperature (i.e., inside a new host). Those bacteria under stress should conserve or express factors that may favor subsequent colonization or infection. We want to test this hypothesis by passing stressed cells into a favorable environment which simulates a new host.

## Materials and methods

### Bacterial strains

Four *A*. *baumannii* clinical isolates were used in this work. All clinical isolates were obtained from different patients (standard service of routine) at the Hospital Universitario Marqués de Valdecilla. HUMV-1319 and HUMV-3743 were isolated from wound exudate, HUMV-2471 was isolated from sputum and HUMV-2790 was isolated from skin ulcer. Reference strain *A*. *baumannii* ATCC^®^ 19606^T^ was also included. *A*. *baumannii* strains were routinely cultured on blood agar (BA) plates, or Luria broth (LB) at 37°C and stock cultures were frozen at -80°C with 20% (vol/vol) glycerol. For inocula preparation, strains were grown at 37°C in LB with shaking (175 rpm) for 24 h. Cells were collected by centrifugation (1.258 g for 20 min) and washed three times with sterile saline solution (0.9% wt/vol NaCl). Finally, cell pellets were suspended in sterile saline solution. *Escherichia coli* DH10B and BL21 strains were used as positive controls for serum susceptibility.

### Bacterial growth curves

Growth curves were carried out to confirm that the strains used in our study grew at similar growth rate. Bacteria from overnight cultures were diluted 1,000 folds in LB and were then inoculated into each well of a 96-well polystyrene flat-bottom microtiter plates and incubated at 37ºC for 24 h inside an Infinite® 200 microplate reader (Tecan). The plates were read every 30 minutes for 24 h at wavelengths of 600 nm with shaking before each cycle.

### Long-term survival assays

*A*. *baumannii* cells from stationary phase were incubated at 22ºC in liquid or solid environments. Starvation was implemented by incubating cells in sterile saline solution or onto sterile cotton white lab coat fragments (~1 cm^2^), plastic (bottom of wells in 24-well plates (1.9 cm^2^, Corning^TM^ Costar^TM^, Fisher Scientific)), or sterile glass cover slides (12 mm diameter, 1.13 cm^2^). For survival assays onto solid surfaces, glass cover slides, plastic, or white lab coat fragments were inoculated with 50 μl spots of *A*. *baumannii* at a cell density of ~10^7^ cells ml^-1^. Cover slides and white lab coat fragments were sterilized by autoclaving (121ºC/20 min) and 24-well plates were purchased from Fisher Scientific (individually wrapped, sterilized by gamma irradiation). For survival assays in the aqueous environment, experiments were carried out in 50 ml polypropylene tubes (Fisher Scientific) containing 15 ml of sterile saline solution reaching a bacterial density of ~10^7^ cells ml^-1^.

Solid environments (cover slides, fragments of white lab coats, and plastic) were placed in wells of several 24-well plates in the dark. Ambient relative humidity and temperature were measured with a hygrometer/thermometer (Thermo Hygro) and maintained at a relatively low humidity level (54 ± 1.6% humidity) and room temperature (RT) 22 ± 0.2ºC.

Populations from solid surfaces were recovered adding 1 ml of saline solution to a glass or plastic surfaces and scraping off the cells from the bottom using a 200 μl tip. To recover *A*. *baumannii* from the white lab coat, the coat fragments were placed in a 15 ml polypropylene tube with 5 ml of saline solution and vigorously shaken with a vortex.

During long-time survival experiments, samples from solid surfaces and saline microcosms were collected at different time points (days 0, 1, 3, 10, 20, 30, 40, 50, 60), serially diluted in saline solution and used to inoculate Luria agar (LA) or LA amended with sodium pyruvate (0.5% wt/vol) [22, 23] to determine the number of colony-forming units (CFUs). Sodium pyruvate was added directly to LA before autoclaving.

Along the desiccation survival period or long-term starvation in saline, and to determine the regrowth capacity, recovered *A*. *baumannii* cells from solid media were resuspended in 1 ml of LB. For saline, 5 μl of the bacterial suspensions were inoculated into 1 ml of LB. These bacterial cultures were placed in wells of 24-well plates and incubated for 48 h at 37ºC. Visual inspection confirmed the purity of cultures on their colonial morphology after plating and by immunofluorescence staining with a specific antiserum against *A*. *baumannii* [24]. Three long-time survival independent experiments were performed.

### Biofilm formation

Biofilm formation capacity of stressed cells and fresh inocula was estimated after addition of 1 ml of LB medium to all solid surfaces contained on 24-well plates (white lab coat, plastic, and glass) and 5 μl of the bacterial inocula for saline, according to previously described protocols [25]. Briefly, after addition of 1 ml LB medium, the microplates were incubated for 48 h at 37°C without shaking. Planktonic cells were removed, and wells were rinsed three times with distilled water, and the remaining adherent bacteria were stained with 1.5 ml/well of crystal violet (CV) (0.7% [wt/vol] solution) for 12 min. Excess stain was removed by washing with distilled water, and CV was extracted with 1.5 ml of acetic acid (33%). Plates were incubated at RT in an orbital shaker for 1 min at 400 rpm to release the dye into the solution. Then, a sample of 100 μl was transferred to another 96-well flat-bottom plate, and the amount of dye (proportional to the density of adherent cells) was determined at 620 nm using a microplate reader (Multiskan FC; Thermo Fisher). In each experiment, results were corrected for background staining by subtracting the value for crystal violet bound to uninoculated controls. The number of planktonic cells (CFUs) was converted to a logarithmic scale, and normalized biofilms were calculated by dividing the total biofilm value (expressed as the OD_620_) by the bacterial growth for each strain (expressed in CFUs). The biofilm assay was performed three times, with duplicates in each assay.

### Confocal Laser Scanning Microscopy (CLSM)

Bacterial viability during the survival assays on cover slides was determined as previously described [26]. Briefly, bacteria were stained using the BacLight LIVE/DEAD kit (Molecular Probes Inc.) for 20 min at RT in the dark. A series of optical sections were obtained with a Nikon A1R confocal scanning laser microscope; the excitation wavelengths were 488 nm (green) and 561 nm (red), and 500 to 550 nm and 570 to 620 nm emission filters were used, respectively. Images were captured at random with a 40× Plan Apo (numerical aperture [NA], 0.75) objective. Reconstructions of confocal sections were assembled using NIS-Elements software, version 3.2. The volume measurement tool of the NIS-Elements software was used to readily recognize the relative biomass of live (green fluorescence) and dead cells (red fluorescence). The percentage of biomass that was alive and the percentage of biomass that was dead in all z-stack images from representative assays was calculated.

### *Galleria mellonella* killing assays

*G*. *mellonella* caterpillars in the final-instar larval stage (Bichosa, Salceda de Caselas, Galicia, Spain) were stored in the dark and used within 3 days from the day of shipment. Caterpillars (250 ± 25 mg in body weight) were employed in all assays.

Two strains of *A*. *baumannii* were arbitrarily selected (HUMV-2790 and HUMV-1319) after starvation in saline solution (60 days). Bacterial suspensions were adjusted to ~10^7^ CFUs ml^-1^, and the same bacterial suspension was prepared from a fresh culture of these strains. Bacterial infection of *G*. *mellonella* was carried out primarily as described by Peleg et al. [27]. Briefly, a 10^-l^ Hamilton syringe was used to inject 10 μl aliquots of the inocula into the hemocoel of each caterpillar via the last left proleg. Ten *G*. *mellonella* larvae were injected with ~10^5^ bacteria and were placed in a 9.0 cm Petri dish lined with 8.5 cm Whatman paper, then incubated at 37ºC in the dark.

Bacterial colony counts on LA were used to confirm all inocula. Larvae were individually examined for melanization, and time of death was recorded. Caterpillars were considered dead when they displayed no movement in response to touch. Assays were allowed to proceed for only 4 days as pupa formation could occasionally be seen by day 4. Two independent replicates of each infection experiment were performed per infection strain. Two negative control groups were always prepared: one group that underwent no manipulation to control for background larval mortality (no manipulation control) and one group (uninfected control) that was injected with saline solution to control for the impact of physical trauma.

### Serum bactericidal assays

All studies involving human samples were performed following international standards for research ethics and were approved by the local institutional review board (Hospital Universitario Marqués de Valdecilla). Human sera were isolated from whole venous blood obtained from healthy human volunteers after informed consent. Venous blood was drawn aseptically and allowed to clot, and the serum was separated by centrifugation. Complement was inactivated by heating serum at 56°C for 30 minutes when required. Two different final serum concentrations (25% and 50%) of normal (N) and inactivated (I) human serum (prepared in PBS) were used against 2 clinical strains maintained for 60 days under starvation (HUMV-1319 and HUMV-2790). CFUs were determined at 0, 30, 90 and 180 min by serial dilution and cultured in LA plates. Experiments were performed three times.

### Scanning Electron Microscopy (SEM)

Bacterial presence in white lab coats was analyzed qualitatively using scanning electron microscopy. White lab coats fragments infected with *A*. *baumannii* inside 24- well plates at time 1 h or after 60 days were processed directly inside the plates. The entire wells were fixed with ice-cold 3% glutaraldehyde for 20 min at 4°C. Samples were then dehydrated in a graded ethanol series, cut into small pieces, dried by the critical point method, coated with gold in a Fine Coat ion sputter (JFC-1100; JEOL), and observed with an Inspect S microscope (FEI Company) working at 15 or 20 kV. Uninoculated autoclaved white lab coat fragments were used as the control for the presence of bacteria.

### Statistics

Data were described with means and Standard deviation and median and interquartile range when appropriate. Dichotomous variables were described with percentages. Comparisons of the quantitative data was carried out by comparing means with the paired Student t-test. The alpha error was set at 0.05, and all *p* values were bilateral. In addition, for dichotomous variables, survival curves were obtained and equality of survival distributions was tested by using the Log Rank (Mantel-Cox) test. We conducted all statistical analyses using Microsoft® Excel version 16.14.1. *G*. *mellonella* mortality curves were plotted using the Graph Pad Prism version 7.0a.

## Results

**Fig 1:**
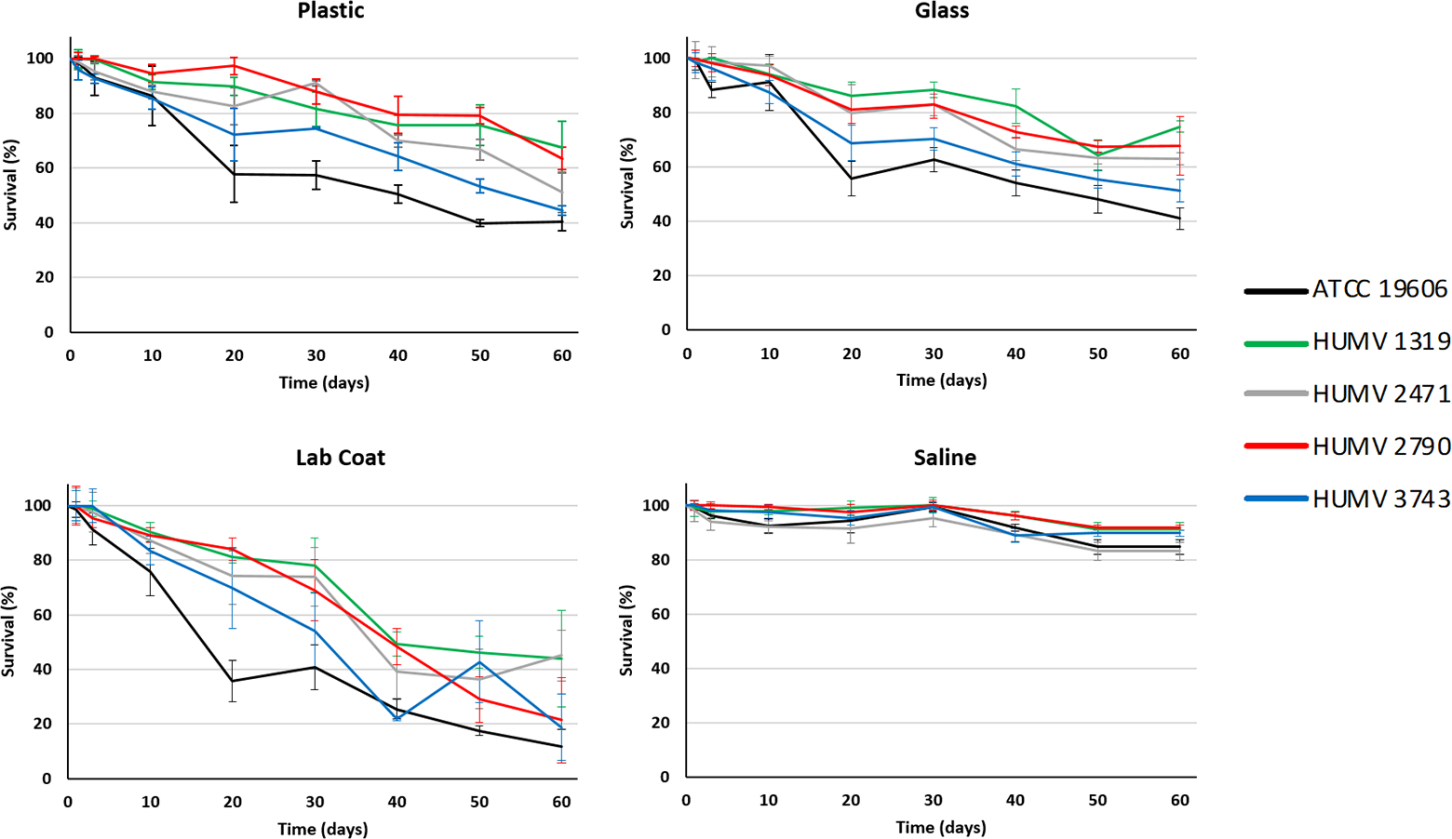
Survival of *A*. *baumannii* strains in different environments. Survival times of strains inoculated onto white lab coat, plastic, or in glass coverslips (54% relative humidity), or suspended in sterile saline solution, and kept at room temperature. Colony counts on LA determined cell survival. Each point represents the mean of three independent experiments expressed as a reduction in culturability with respect to day 0 (100%). Error bars show the standard error of the mean.

The effects of time (60 days period) and nutrient deprivation upon culturability of *A*. *baumannii* populations on solid surfaces and saline solution are shown in Fig 1. On average, *A*. *baumannii* culturability was reduced by 46.66%, 40.40%, 71.78%, and 11.94% in plastic, glass, white lab coat, and saline, respectively. The less resistant strain on solid surfaces was the reference strain ATCC^®^ 19606^T^ where its culturability was reduced in 59.68%, 58.95%, and 88.11% in plastic, glass, and white lab coat, respectively. The most resistant strain on plastic and glass was HUMV-1319, with survival rates of 67.51% and 74.76%, respectively. Strain HUMV-2471 was the most persistent on white lab coat fragments, with a reduction of 54.88% in culturability on this surface after 60 days, indicating that near half the population of this strain survive on a white lab coat for at least two months.

The effect of starvation on bacterial populations maintained under starvation in saline solution was minimal. In all cases, we observed that strains kept survival rates above 83%, and did not change throughout the experiment’s duration.

When colony-forming units were enumerated on LA supplemented with sodium pyruvate (recovery medium), no significant differences were found (p>0.05, data not shown).

We tested the capability of the starved bacterial survivors to resume growth and to express adherence factors to form biofilms by CV assays. Despite a reduction in culturability over 60 days, some populations recovered from starvation on solid surface experiments and retained their ability to regrow and to form biofilms after rehydration with culture medium (S1 Fig).

**Fig 2:**
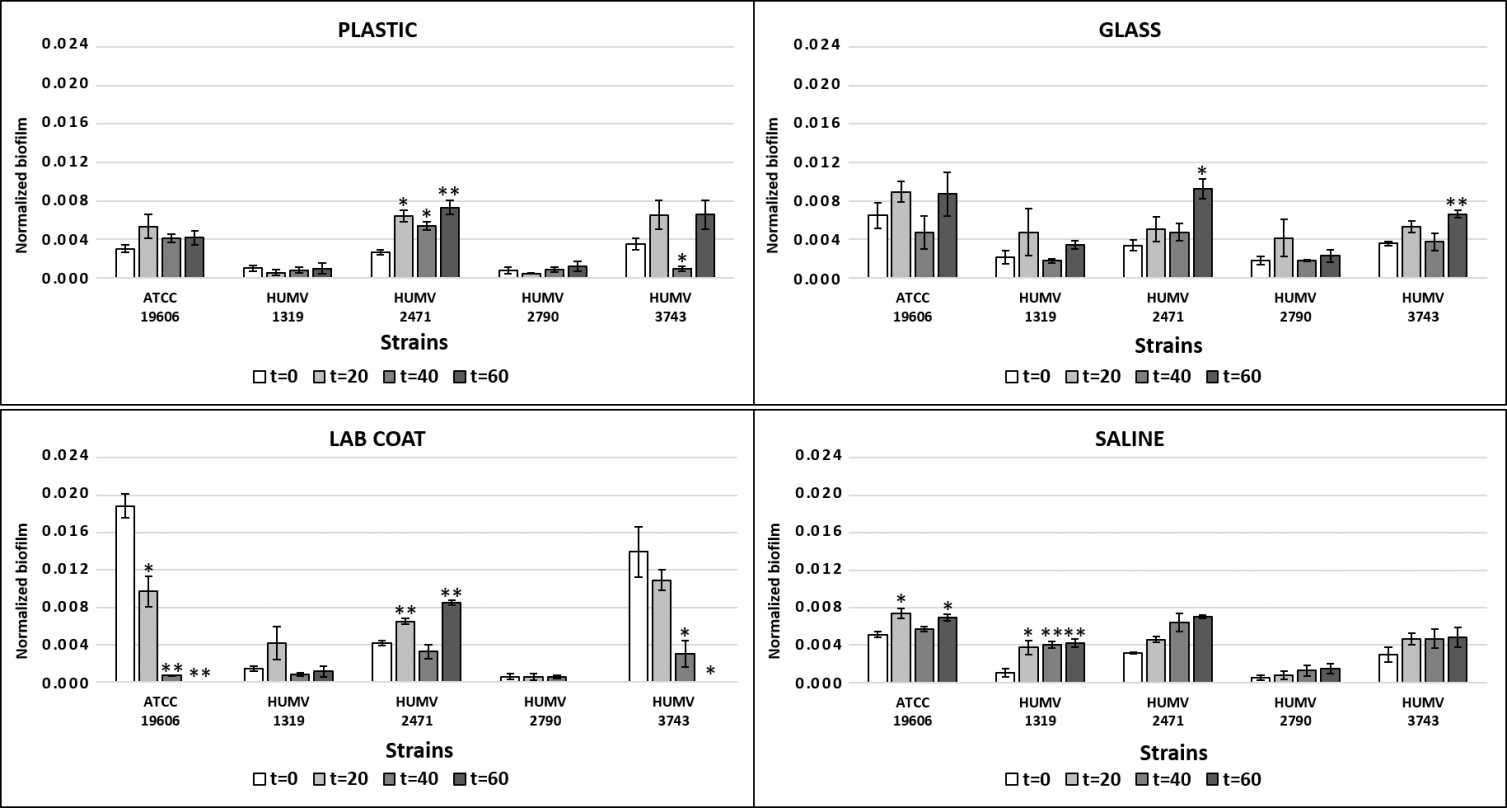
Quantification of biofilm formation. Biofilm formation by *A*. *baumannii* strains after desiccation and rehydration with LB. Quantification of biofilm formation was performed after crystal violet extraction and measurement (OD_620_). Normalized biofilm formation, calculated as the total biofilm (expressed as the OD_620_) divided by growth (expressed in CFUs). Time is indicated in days. Values are presented as the mean ± standard error (SE) of three independent experiments. Asterisks indicate: *, p<0.05; **, p<0.01.

The number of CFU was converted to a logarithmic scale, and normalized biofilms were calculated by dividing the total biofilm value (expressed as the OD_620_) by the bacterial growth for each strain (expressed in CFUs) (Fig 2).

Growth curves confirmed that the strains used in our study grew at a similar growth rate (S2 Fig). Our results indicate that some strains retain, or even increase, their ability to form biofilms after rehydration.

**Fig 3:**
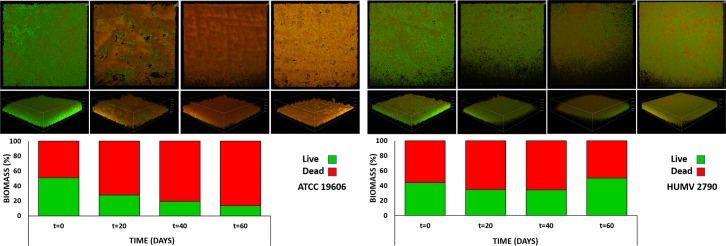
Confocal Laser Scanning Microscopy of live/dead cells. CLSM images of two *A*. *baumannii* strains after survival onto glass coverslips at different times (up to 60 days). Representative examples of strain *A*. *baumannii* ATCC^®^ 19606^T^ (the worst survivor, left), and of HUMV-2790 (the best survivor, right) are shown. Bacteria were stained with the BacLight LIVE/DEAD viability kit. Live cells fluoresce in green with Syto 9 dye, and dead cells are stained red with propidium iodide. Original magnification: ×400. Lower panel: fluorescence (live/dead) for each strain represented in the upper panel, expressed as a percentage.

Staining of *A*. *baumannii* strains spotted on the glass by using the BacLight LIVE/DEAD bacterial viability kit demonstrated the transition from mostly viable populations (0 days, most of the cells fluoresced in green) to populations where cells with compromised cytoplasmic membranes predominated (red fluorescent cells) (Fig 3). A more precise quantification using reconstructions of confocal sections by the NIS-Elements software reveals fewer differences between live and dead bacteria at early time-points, but overall, the presence of a fraction of cells with intact cell membranes (green fluorescent cells) could be detected after 60 days. Moreover, the time required for the loss of membrane integrity differed between strains. After 20 days of desiccation, a significant proportion of the cells of the strain ATCC^®^ 19606^T^ fluoresced in red (Fig 3 left) while most of the populations of the clinical isolates remained viable, in some cases, up to 60 days (Fig 3 (right) and S3 Fig).

**Fig 4:**
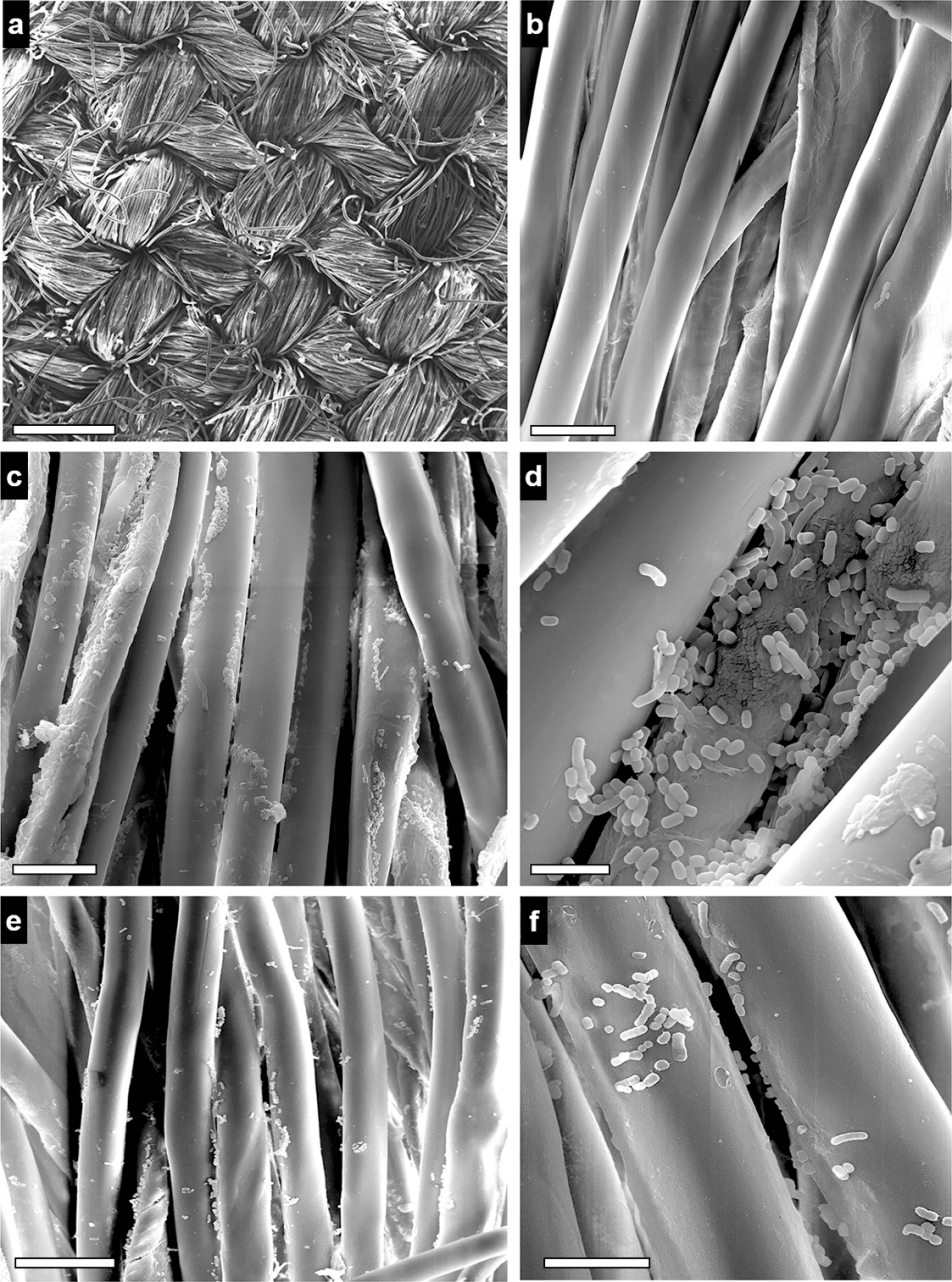
SEM analysis. Scanning electron microscopy analysis of the morphology of *A*. *baumannii* (strain ATCC^®^ 19606^T^) cells maintained on white lab coat fragments for up to 60 days. Panel shows random microscopy fields observed at different magnifications. a,b, control samples, and c,d, infected samples at the beginning of the experiments (day 0). e,f, infected samples after 60 days at 22ºC. Original Magnification: a, ×100; b,c,e, ×2.500; d, ×10.000; f, ×5.000. Scale bars: a, 0,5 mm; b, 20 μm; c,e, 25 μm; d, 5 μm; f, 10 μm.

We examined white lab coat fragments inoculated with bacteria by scanning electron microscopy to study the morphology of bacterial adherence on that surface. SEM revealed some cells with slightly altered cell morphology after long-term starvation and desiccation (Fig 4E and 4F) in comparison with fresh samples (Fig 4C and 4D). Bacteria seem to be firmly attached to the lab cotton fibers even after several washing steps and a series of ethanol washes during the sample processing.

**Fig 5:**
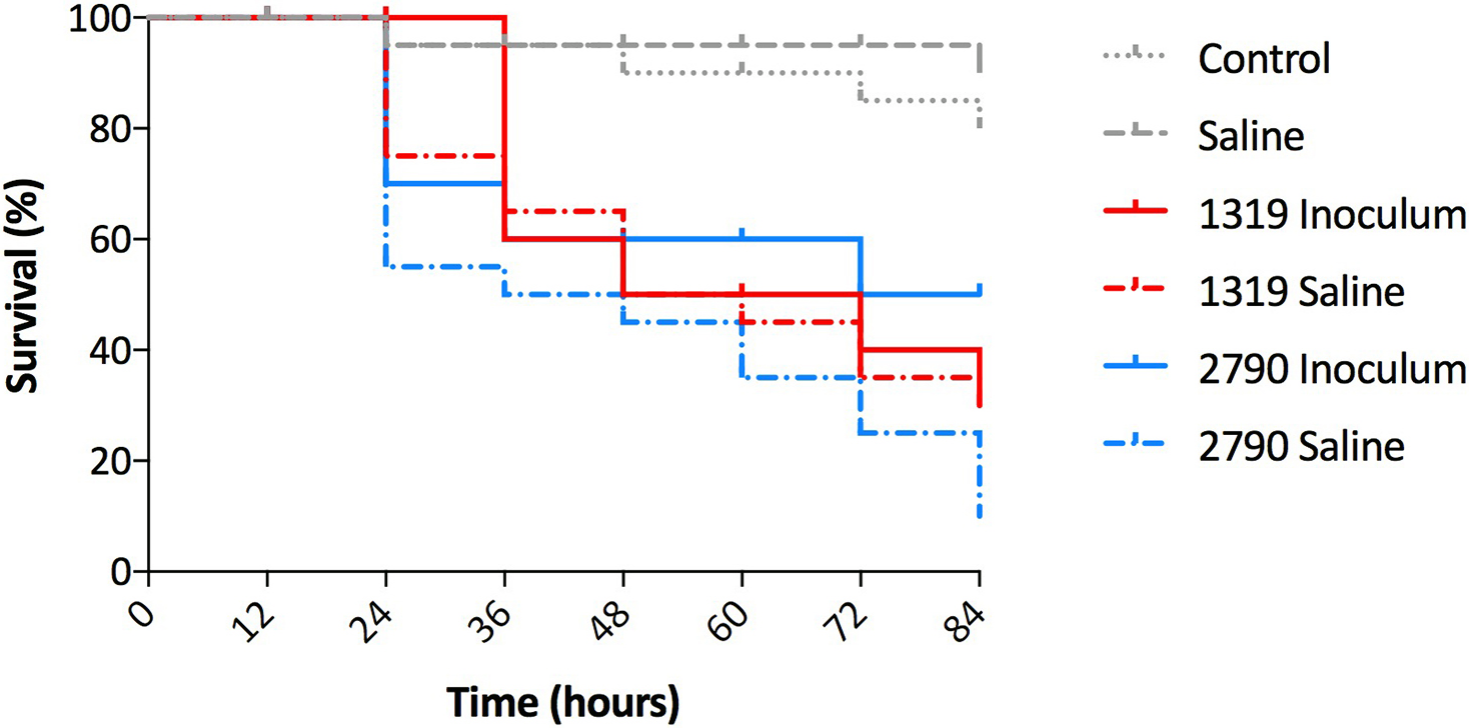
*Galleria mellonella* killing assays. Survival rate of worms after challenge with two *A*. *baumannii* strains. Ten larvae were infected with saline alone, with 10^5^ CFU of each strain or uninoculated (no manipulation control), incubated at 37°C for 84 h and the time of the death of the larvae was recorded. Results are the mean of two separate experiments.

*G*. *mellonella* larvae were challenged with fresh inocula of two *A*. *baumannii* strains and compared with larvae challenged with stressed bacteria (prepared from saline suspensions) of the same strains. Survival was recorded every 12 h for up to 84 h. 10^5^ CFUs (per larvae) from strains HUMV-2790 and HUMV-1319 killed 50% of larvae after 72 h, and 50% and 70% of larvae after 84 h, respectively. Nevertheless, these strains killed more than 60% of larvae after 72 h after having suffered stress. Moreover, stressed strain HUMV-2790 killed 90% of larvae after 84 h (Fig 5).

**Fig 6:**
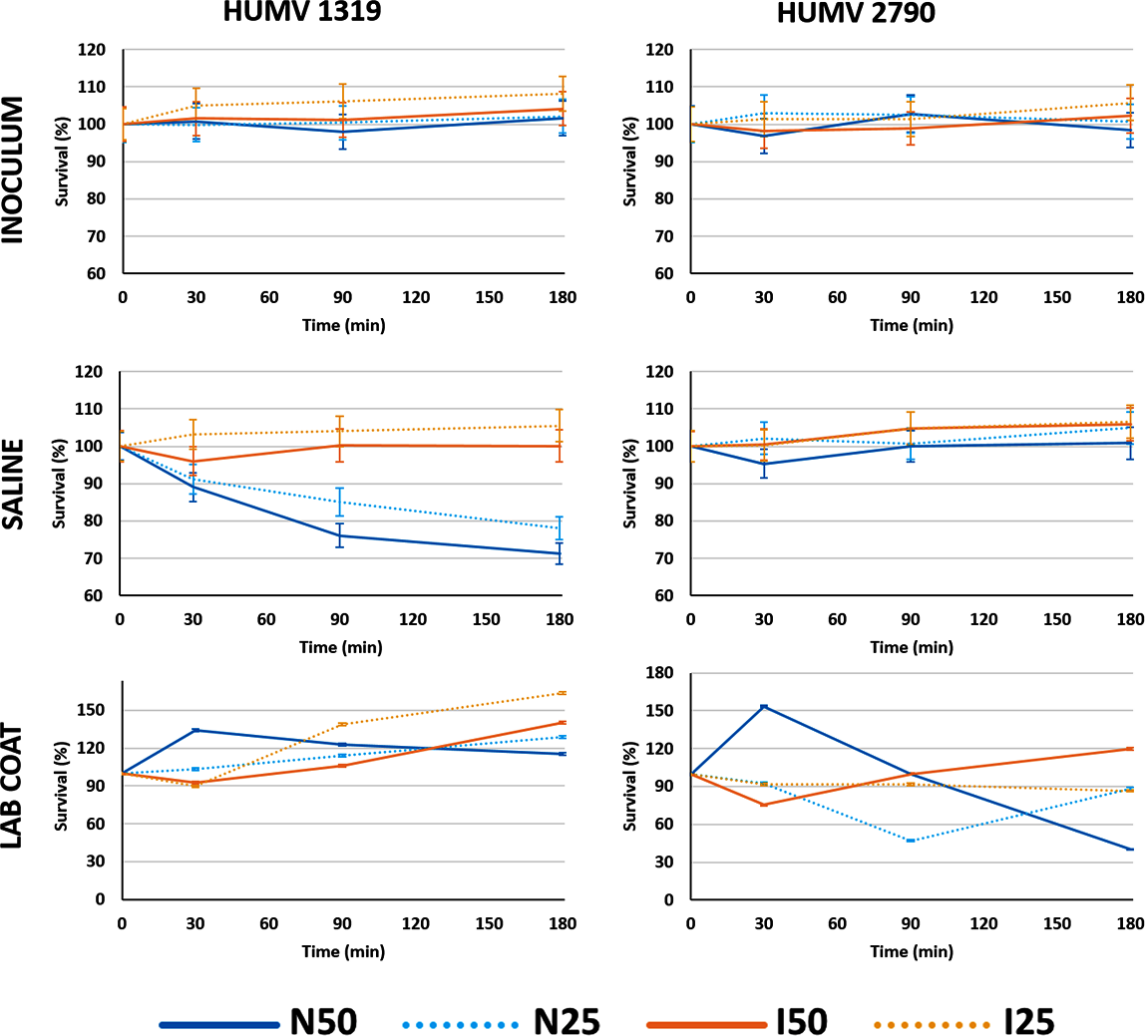
Effect of stress conditions on *A*. *baumannii* survival in human serum. Survival ability of *A*. *baumannii* strains from fresh cultures or different stress conditions in 50% (N50, solid lines) and 25% (N25, dotted lines) non-immune human serum (blue) or in inactivated serum (orange). Results are presented as percentage survival relative to 100% of the initial inoculum. Values shown are means of three replicates from three independent experiments.

To analyze the contribution of stress adaptation to human serum resistance or sensitivity, we tested strains HUMV-2790 and HUMV-1319 in a serum survival assay. Inocula from these strains were prepared from populations maintained in liquid environments or onto lab coat fragments and compared with fresh cultures (inoculum). Both strains were resistant to human serum when suspensions were made from LB medium. After long-term starvation, both strains were also resistant when inocula from lab coat fragments were tested. Strain HUMV 1319 shows a reduction in viability when bacteria came from saline and was resuspended in normal serum but not in inactivated serum (unpaired T-test, p = 0.002). Conversely, strain HUMV 2790 grows well in both normal and inactivated serum (Fig 6). As expected, *E*. *coli* strains were susceptible to human serum (S4 Fig).

## Discussion

The survival of pathogenic microorganisms in the healthcare environment has a significant role in nosocomial infections. Patient contamination may result from healthcare workers’ hands, medical devices, or also by the direct patient shedding of bacteria, which can survive up to several months on inanimate surfaces [28]. Since different surfaces may influence the survival times, we used materials that are typical for the hospital environment to study the survival ability of *A*. *baumannii* strains, including white laboratory coats used by professionals in the medical field and those involved in laboratory work. According to several authors, *A*. *baumannii* can survive desiccation, although this ability varies dramatically depending on the strains tested [18, 21, 29, 30]. Our results show that the long-term survival of *A*. *baumannii* on dry surfaces was only slightly affected by the material used because a constant reduction in culturability was observed onto plastic, glass, and lab coat surfaces. In general, the resistance to stressful conditions was high, being the reference strain the most susceptible to desiccation. Remarkable is the survival of all strains on the white lab coat fragments. In a recent study, Munoz-Price found concordance between contamination of hands of healthcare workers and their white lab coats [31] and their results include strains of *Acinetobacter* spp. as contaminants.

Staining of *A*. *baumannii* strains spotted on glass demonstrated the presence of cells with intact cell membranes after long-term starvation and desiccation. Again, reference strain ATCC^®^ 19606^T^ was the most sensitive to these conditions. Viable but nonculturable state was not induced in *A*. *baumannii* under these conditions because adding sodium pyruvate to the recovery medium does not affect culturability [22, 23]. Our SEM analysis also confirmed that some strains remain attached to cotton fibers on the white lab coat fragments after long-term starvation and desiccation.

Our results correlate with those of Bravo et al. with *A*. *baumannii* populations starved in a liquid environment and with our previous findings using *A*. *pittii* strains, a less well-known species of the genus *Acinetobacter* [26].

Among the responsible mechanisms that could allow nosocomial pathogens to persist with these stress conditions are their ability to resist desiccation and to form biofilms. The ability of *A*. *baumannii* to form biofilms is a potential virulence factor that has received some attention. However, although adherence is a prerequisite for infection, *A*. *baumannii* shows low adherence to epithelial cells [24]. In this work, we used three biofilm-forming and two non-biofilm-forming strains. Importantly, biofilm-forming strains retain or even increase their capacity to form biofilms after rehydration, despite a considerable reduction in culturability over time.

*A*. *baumannii* must first evade serum bactericidal activity to establish infection. Several studies show that a significant proportion of clinical *A*. *baumannii* strains are resistant to killing by normal human serum [32–34]. We wanted to know if long-term starvation may reduce *A*. *baumannii* resistance to human serum, which could help the immune system to fight opportunistic infections. Our results show that bacteria, despite being under stress for a long time, retain their capacity to resist and even to grow in human immune serum. Interestingly, one strain seems to reduce its capacity for growth in 50% serum after being a long time on the cotton surface.

Following with virulence traits, we wanted to know if *G*. *mellonella* larvae can be killed by *A*. *baumannii* stressed cells. Testing virulence of strains from lab coat experiments would be also interesting but we have not obtained enough bacteria to perform the *Galleria* assays. Using strains maintained for 60 days under starvation (saline), when compared with their respective fresh inocula no loss of virulence was observed in the *Galleria* model. This suggests a rapid adaptation of bacteria to the temperature shift (from room temperature to 37ºC) and to the availability of nutrients (from starvation to food availability), conditions that bacteria can easily find in a new patient. The data presented here contribute to a better understanding of the resilience and risk of *A*. *baumannii* in hospital settings.

Although more than 80 full genome sequences of *A*. *baumannii* have been published, very few potential virulence factors have been identified. We have recently shown that *A*. *baumannii* can escape from macrophages but is easily eliminated by other human immune cells [35]. This means that *Acinetobacter* could merely perform its job by resisting the serum bactericidal activity, multiplying in blood and releasing its potent stimulator of the immune response, the lipopolysaccharide.

## Supporting information

S1 Fig Biofilm formation by *A*. *baumannii* strains after desiccation and rehydration with LB medium. Shown are representative examples of biofilm formation in 24-well plates by the 5 *A*. *baumannii* strains spotted onto various surfaces after rehydration and growth in LB medium for 48 h at 37°C. Wells were stained with crystal violet.
(TIF)

S2 Fig Growth curves of *A. baumannii* strains growing in 96-well plates in Luria broth. Values are means of bacterial density measured at OD_600_. Bars indicate mean±SD of four independent replicates.
(TIF)

S3 Fig Confocal Laser Scanning Microscopy of live/dead cells. Representative examples of CLSM images of three *A*. *baumannii* strains after survival onto glass cover slips at different times (up to 60 days). Bacteria were stained with the BacLight LIVE/DEAD viability kit. Live cells fluoresce in green with Syto 9 dye and dead cells are stained red with propidium iodide. Original magnification: ×400. Lower panel: fluorescence (live/dead) for each strain represented in the upper panel, expressed as percentage.
(TIF)

S4 Fig Effect of human serum on a susceptible bacterium. *Escherichia coli* DH10B **a)** and BL21 **b)** strains were used as positive controls for serum susceptibility in 50% non-immune human serum (blue) or in inactivated serum (orange). Results are presented as percentage survival relative to 100% of the initial inoculum. Values shown are means of three replicates from three independent experiments.
(TIF)
