# Supplementary figures and images for: Acinetobacter baumannii maintains its virulence after long-time starvation
Source: PLoS One. 2018 Aug 22;13(8):e0201961. doi: 10.1371/journal.pone.0201961 (PMC6104976; doi:10.1371/journal.pone.0201961)

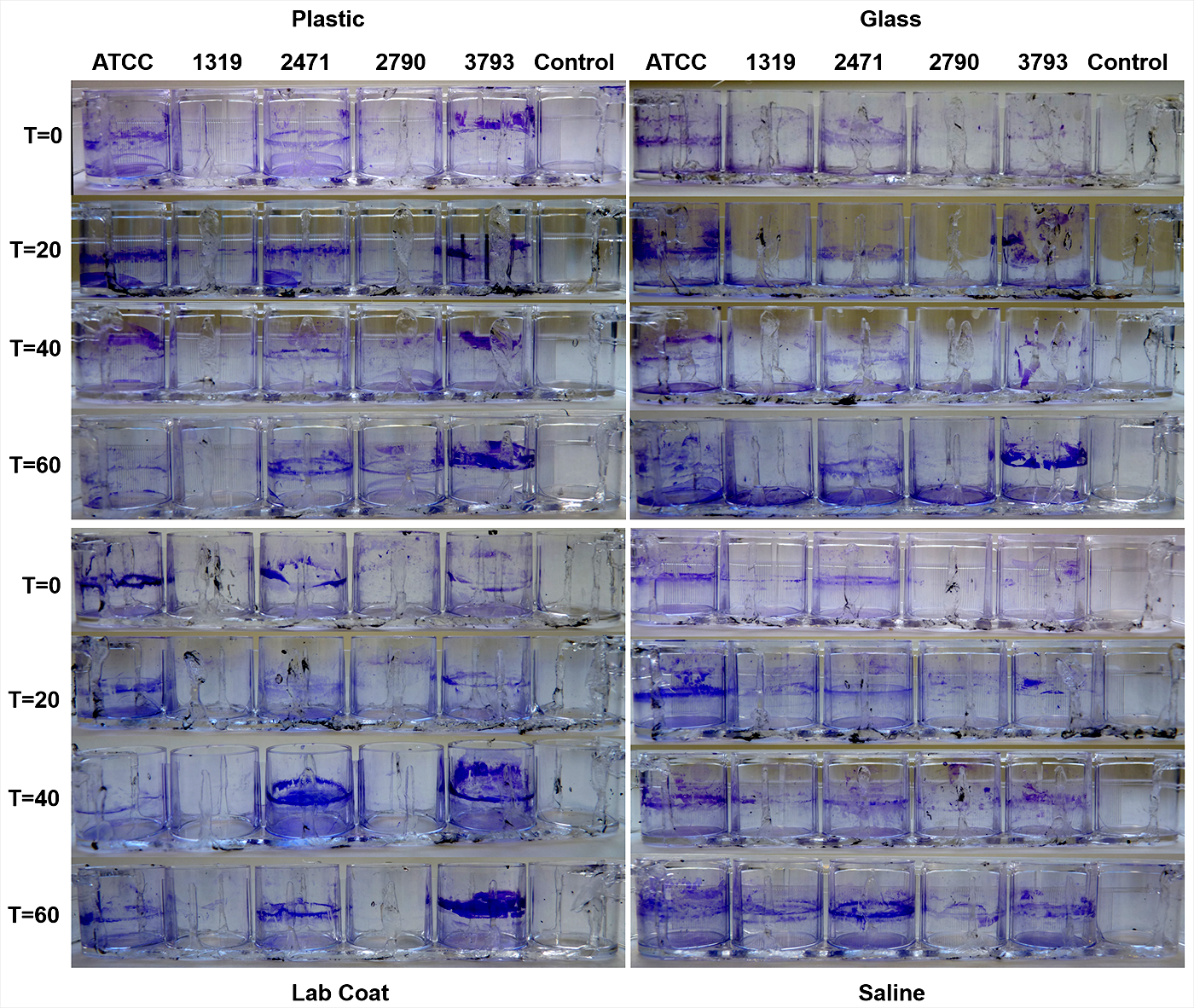

Supplement: S1 Fig — Shown are representative examples of biofilm formation in 24-well plates by the 5 A. baumannii strains spotted onto various surfaces after rehydration and growth in LB medium for 48 h at 37°C. Wells were stained with crystal violet. (TIF) [file pone.0201961.s001.tif]

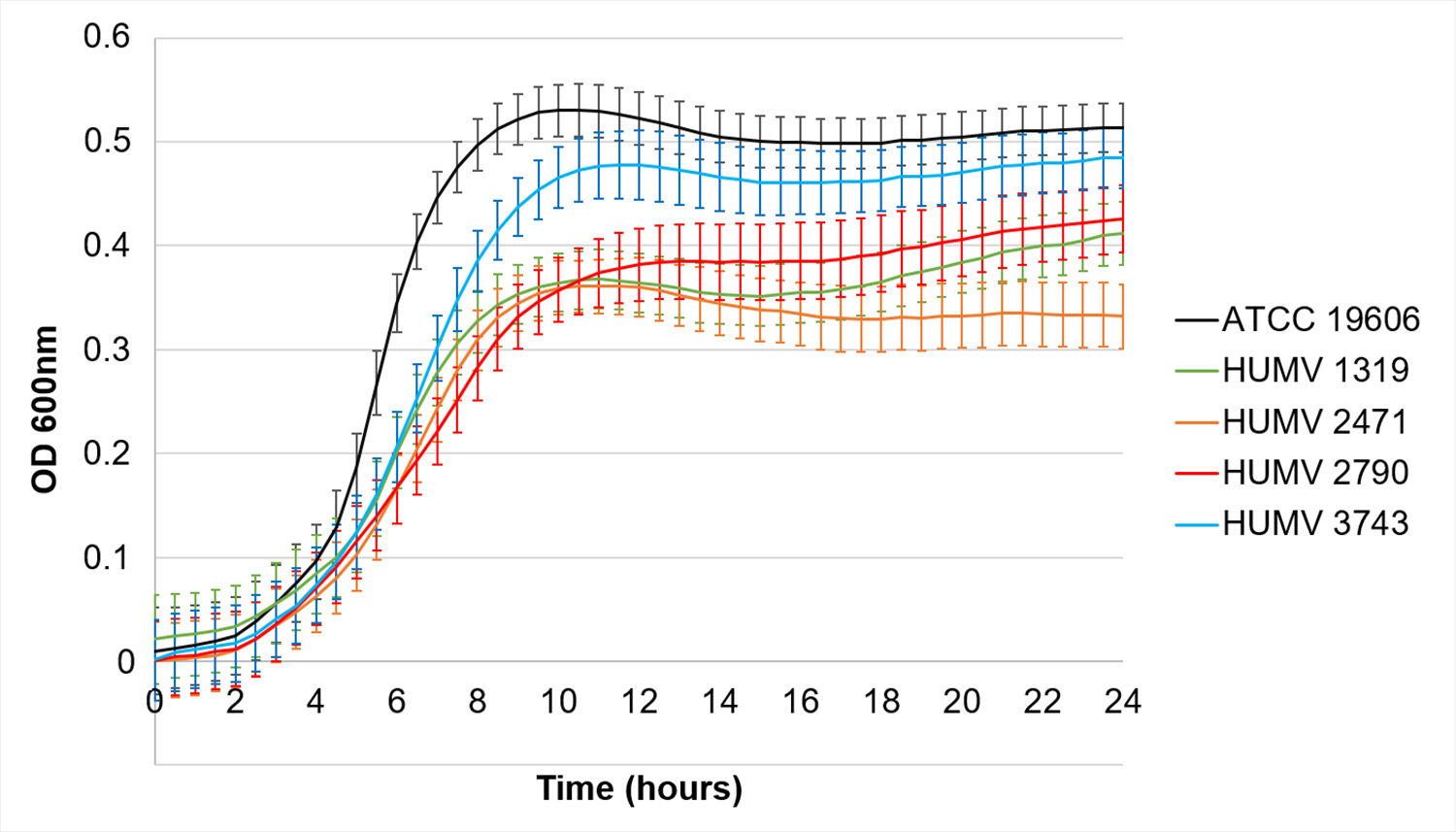

Supplement: S2 Fig — Values are means of bacterial density measured at OD600. Bars indicate mean±SD of four independent replicates. (TIF) [file pone.0201961.s002.tif]

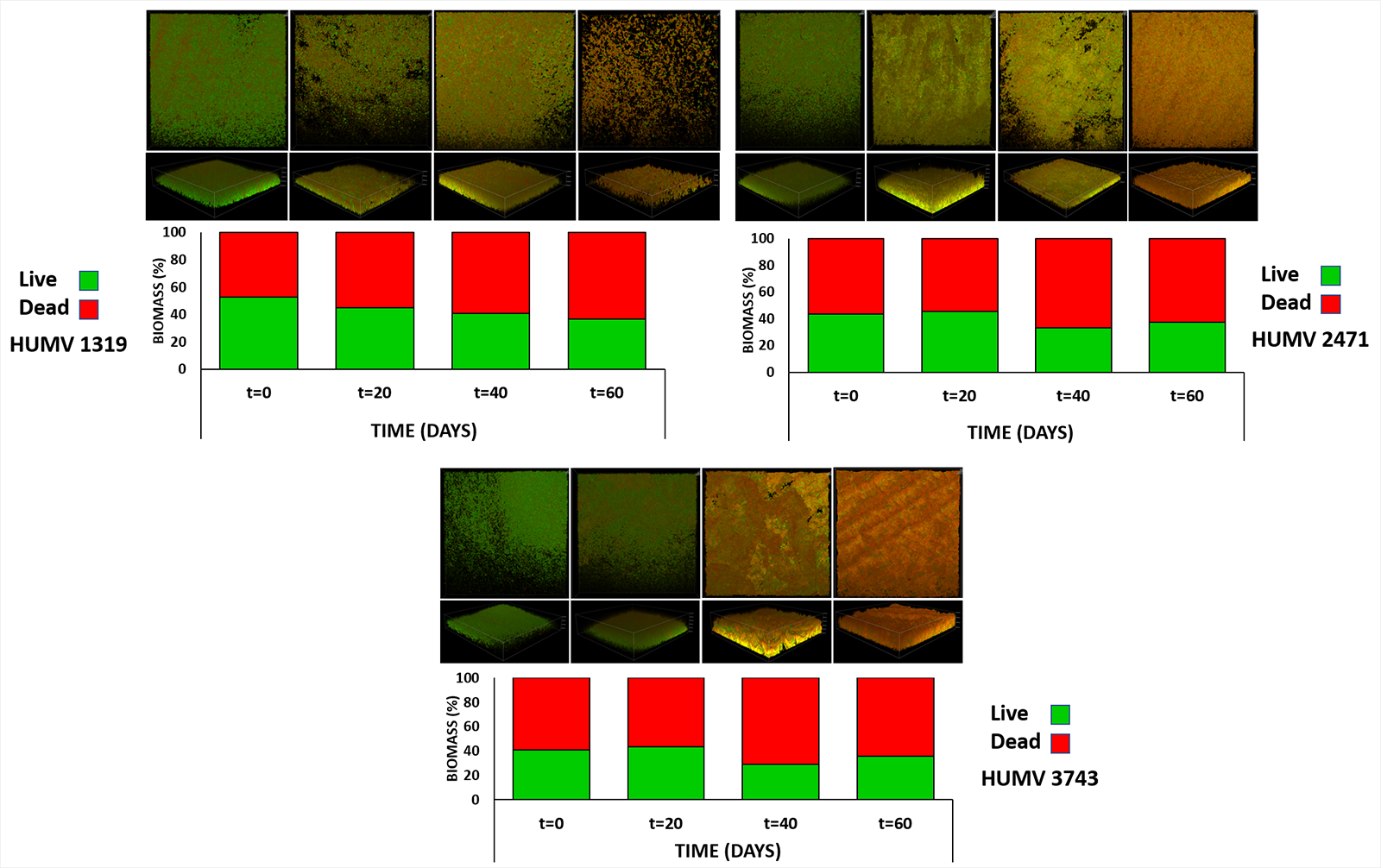

Supplement: S3 Fig — Representative examples of CLSM images of three A. baumannii strains after survival onto glass cover slips at different times (up to 60 days). Bacteria were stained with the BacLight LIVE/DEAD viability kit. Live cells fluoresce in green with Syto 9 dye and dead cells are stained red with propidium iodide. Original magnification: ×400. Lower panel: fluorescence (live/dead) for each strain represented in the upper panel, expressed as percentage. (TIF) [file pone.0201961.s003.tif]

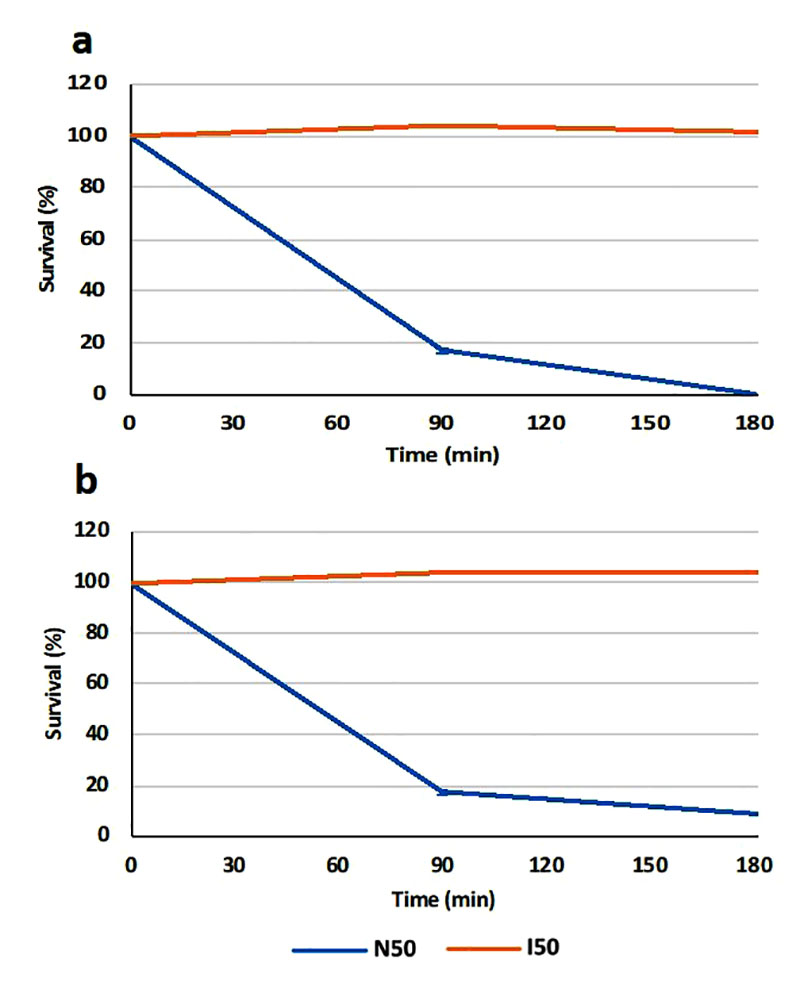

Supplement: S4 Fig — Escherichia coli DH10B a) and BL21 b) strains were used as positive controls for serum susceptibility in 50% non-immune human serum (blue) or in inactivated serum (orange). Results are presented as percentage survival relative to 100% of the initial inoculum. Values shown are means of three replicates from three independent experiments. (TIF) [file pone.0201961.s004.tif]
